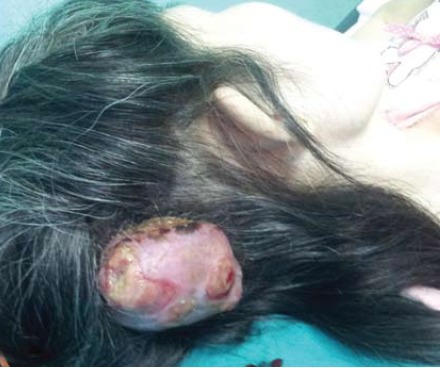# Rare Presentation of Metachronous Multicentric Pelvic and Extracranial Chondrosarcoma : A Case Report

**DOI:** 10.5704/MOJ.1407.006

**Published:** 2014-07

**Authors:** CYL Choong, HZ Chan, A Azuhairy, M Anwar Hau, O Zulkiflee

**Affiliations:** Department of Orthopaedics, Hospital Pulau Pinang, Georgetown, Malaysia; Department of Orthopaedics, Hospital Pulau Pinang, Georgetown, Malaysia; Department of Orthopaedics, Hospital Pulau Pinang, Georgetown, Malaysia; Department of Orthopaedics, Hospital Raja Perempuan Zainab II, Kota Baru, Malaysia; Department of Orthopaedics, Hospital Pulau Pinang, Georgetown, Malaysia

## Abstract

Conventional chondrosarcomas rarely metastasize and it is extremely unusual to see multicentric- behaviour in malignant cartilage tumour. We report a 40 year old lady with presentation of two non-contiguous metachronous foci of low to intermediate grade of chondrosarcoma over left pelvic bone and right scalp respectively in the absence of pulmonary or visceral metastasis.

**Key Words:**

vacuum assisted closure, infection, instrumentation, spine

## Introduction

Chondrosarcoma is the third most common primary malignant tumour of bone, following Ewing’s sarcoma and osteosarcoma ^4,5^. It constitutes approximately 20 % of all primary malignant bone neoplasms ^3,4^. There is higher preponderance in axial than the appendicular skeleton with the pelvis being the most common site, accounting for about 40% of all reported cases ^3^. The spine and the craniofacial bones are very rarely involved. Although metastasis is rare with conventional chondrosarcoma, it can metastasize to lungs, liver and other bones ^1^. It is even rarer for chondrosarcoma to metastasize to extraosseous regions, like the brain as accounted by Faris et al ^2^. In addition, it is also well-known that multicentricity, which is defined as the presence of two or more separate chondrosarcomas in the absence of visceral involvement between the time of diagnosis of both tumours, is extremely unusual in malignant cartilage tumours, except for the highly aggressive mesenchymal chondrosarcoma

To our knowledge, we have encountered a case of multicentric chondrosarcoma with metachronous involvement of pelvic bone and scalp (extra-osseous), a feature that has been reported previously.

## CASE REPORT

A 40 year old female, presented with a painless left hip swelling for six years prior to presentation. She had first noticed the swelling in 2008 and sought treatment at a private hospital, but defaulted treatment for six years until two weeks prior to hospital admission, when she developed left hip pain which radiated to the left knee, and had walking difficulties. The patient appeared cachexic and pale. Physical examination revealed a painless huge bony mass, measuring about 20 x 15cm arising from her left hip extending over the suprapubic region and beyond the midline of the stomach. It had ill-defined margins and the range of movement in the left hip was limited. Otherwise, distal pulses were present normally with no neurological deficit over the left leg. No lymph nodes were detected.

MRI findings showed a huge lobulated intermediate to high T2 signal intensity mass in the left iliac fossa arising from the pelvis, measuring 165 x 80 x 210 mm. The mass extended from the level of iliac crest down to upper third left femur crossing the midline to the right iliac fossa. There was involvement of the left ilio-psoas muscle with destruction of the anterior column of the left acetabulum. The left femoral vessel was encased within the mass. Otherwise, the outline of the mass was fairly well circumscribed. There was no infiltration of the urinary bladder or uterus. The left femoral head was intact. CT scans of the thorax, abdomen and pelvis showed no evidence of distant metastasis.

Trucut biopsy of the mass showed abundant hypocellular, avascular, hyaline chondroid matrix separated by fibrocollagenous septa into lobules. Embedded within the matrix were malignant chondrocytes located within lacunae . These chondrocytes exhibited mild to moderate nuclear pleomorphism, with occasional binucleation and low mitotic activity. No tumour necrosis was evident. These findings were consistent with well-differentiated chondrosarcoma.

**Fig. 1A. Chondrosarcoma of the left pelvis extending into left thigh; B. Pelvic tumour measuring 220mm x 150mm and weighing 2100g:**
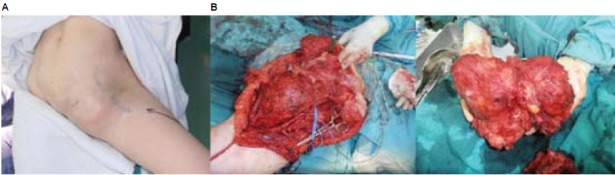


A multi-disciplinary intervention by the orthopaedic, surgical, radiological, anaesthetic and oncology team was adopted. She underwent pelvic resection Type II (periacetabular), Type III (os pubis, ischium) and partial Type I (iliac) as classified by the Musculoskeletal Tumour Society. The resection revealed the extensive growth of the tumour. A large 220mm x 150mm mass, weighing at 2100g was resected from the pelvis (Figure 1B). The invading tumour was found to be extending from the inner part of the left ischium, the lower part of the left ilium beyond the midline and the inner part of left pubic bone down to medial part of the left thigh. Pelvic structures, namely the bladder and uterus, were compressed to the opposite side and the left common iliac vessels were encased by the tumour. Following the resection, pelvic reconstruction surgery with ilio-femoral fusion was done. Ilio-femoral fusion was performed instead of hemipelvic reconstruction with megaprosthesis due to financial constraints on the part of the patient.

Due to the extensiveness of the tumour, the surgical team had to perform peritoneum and abdominal closure after resection of the tumour. The bladder was noted to be thinned-out and was repaired.

Post-operatively, the patient made an uneventful recovery and was discharged well with wheelchair ambulation. Post-operative histopathological findings of the resected tumour showed narrow surgical clearance of 1mm away from all soft tissue surgical margins except at the lateral margin which was 2mm away. The pelvic bone was invaded by the tumour including the superior margin of the ilium. However, the articular surface of the acetabulum, pubic symphysis margin, pubic rami and femoral head were tumour-free. The pelvic tumour showed well-differentiated chondrosarcoma.

On follow up review of the patient at two months, the patient’s surgical wounds had healed well and she was ambulating with walking frame. Serial radiographs showed bone healing with callus formation at the reconstruction site. However, a painless scalp swelling which was firm in consistency, measuring 3 x 2 cm over the right temporo-occipital region was found (Figure 5). Upon further interrogation, the patient admitted to being aware of the scalp swelling two years after becoming aware of the hip swelling. An ultrasound of the scalp revealed a lobulated hypoechoic lesion within the scalp layer with minimal intralesional vascularity.

An MRI brain showed a well-defined 36 x 31 x 16 mm mixed enhancing lesion in the soft tissue over the right occipital region, with no bone erosion or enhancement noted. No focal lesions were seen in the cerebral hemispheres and cerebellum.

An incisional biopsy of the scalp swelling demonstrated tumour fragments with overlying epidermis lined by benign stratified squamous epithelial cells. The tumour itself appeared lobulated and separated with fibrous septa and composed of large amount of chondroid matrix, with mild cellularity. Malignant chondrocytes were seen within swollen lacunar spaces and the nuclei exhibited moderate nuclear pleomorphism, consistent with well-differentiated chondrosarcoma.

At six months follow-up after the major resection, she presented with a firm mass over the right pubic region. CT scans of the pelvis revealed recurrent chondrosarcoma with extensive bilateral pelvic involvement. There were multiple lobulated rim enhancing hypodense lesions in the soft tissue and muscle around the bones in the pelvis. The lesions were seen on both sides of the iliac bone, proximal portion of adductor and obturator muscles, sacroiliac joints and lateral wall of pelvis, anterior wall of pelvis, perineal region. Large lesions were noted in the right pubic region with destruction of the bone. Some of the lesions extended into the right hemipelvic cavity compressing and displacing the pelvic structures. There were also similar lesions in the course of the external iliac vessels bilaterally, which were likely metastasis to adjacent lymph nodes. Currently, the patient is undergoing palliative management by the oncology team.

## Discussion

Multicentric presentation for skeletal sarcomas is a rare occurrence. With regard to chondrosarcomas, multicentricity is extremely unusual, especially in the non-mesenchymal variants. In our case, this condition was demonstrated by the presentation of two non-contiguous chondrosarcomas, one originating in the left pelvic region and the other originating in the right scalp, without evidence of visceral or pulmonary metastases.

There is much ongoing debate in the literature on whether multicentric chondrosarcomas represent multiple distinct primary lesions or metastatic disease. From the history, we know that the patient had two separate swellings at presentation without evidence of pulmonary dissemination on CT scans. The absence of metastases at presentation supports the possibility that the chondrosarcoma lesions are multicentric and metachronous.

The present case also suggests that when a patient presents with chondrosarcoma, a thorough physical examination of the patient is imperative, during which not only must the primary lesion be examined, but care must also be taken to be vigilant of other secondary lesions because of the reasons as mentioned above. This is especially so during follow-up care so as to detect small insidious lesions that may be masked. As in this case, the patient had a small swelling over her scalp. It had been hidden underneath the thick weaves of her hair. It was only detected when it grew large enough to peek though the thick covering of hair.

Reiner et al conclude that the prognosis and survival rate in patients with pelvic chondrosarcoma is determined by the tumour stage and the surgical margin achieved, where the incidence of local recurrence is influenced by the surgical margin achieved and the incidence of distant metastases is influenced by the tumour stage.5 Early diagnosis is difficult because patients with pelvic chondrosarcoma are usually relatively asymptomatic early in the course of the disease and tend to present at a later stage where the tumour - has already reached a large size. This is because of the wide space within the pelvic cavity that can accommodate a growing tumour without any compressive symptoms until it is large enough to cause mass effect. In this case, the patient had the painless hip swelling for six years before finally seeking treatment only after she started experiencing pain over her left lower limb, incapacitating her ability to ambulate. The patient’s survival rate in this case was further complicated because, due to the extent of the tumour growth, it was impossible to get a wide surgical clearance. Histopathological findings of the surgical resection demonstrated a narrow surgical clearance margin especially over the site of the femoraliliac reconstruction. Unfortunately, six months later, the patient presented with multiple skeletal metastases and extra-skeletal lesion, making any subsequent surgery almost impossible.

In summary, pelvic chondrosarcoma poses a challenge for orthopaedic oncologists because of the often late diagnosis, which results in extensive growth of tumour invading and jeopardizing vital structures and compromising pelvic structure stability. Thus, careful surgical planning and consideration of method of reconstruction is warranted. Long-term follow-up is also necessary in view of the high possibility of local tumour recurrence.

(Figure 2)

(Figure 3)

(Figure 4)

**Fig. 2: Plain radiographs of Pelvis – Anterior posterior (AP) views (right) and Lateral views (left):**
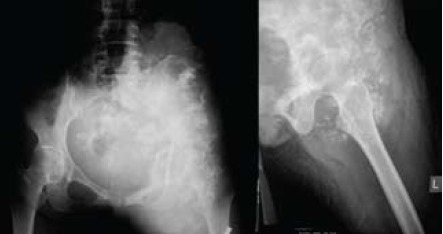


**Fig. 3: MRI scan of pelvis showing pelvic mass within pelvic cavity extending to left thigh:**
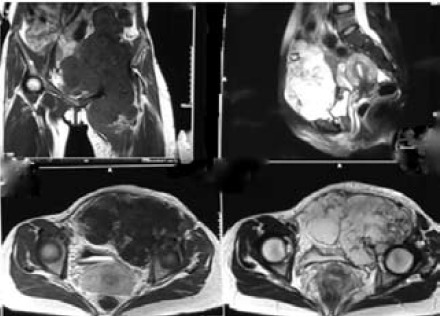


**Fig. 4: Plain radiographs of pelvis (right) and left hip (left) - after -reconstruction:**
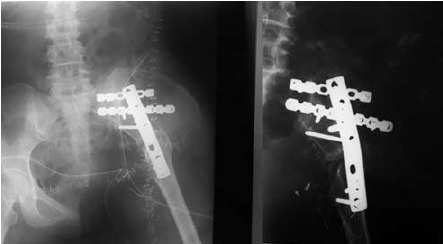


**Fig. 5: Scalp mass over right occipital region:**